# Active surveillance in intermediate risk prostate cancer: is it safe?

**DOI:** 10.1590/S1677-5538.IBJU.2016.03.03

**Published:** 2016

**Authors:** G. van der Poel Henk, C.N. van den Bergh Roderick

**Affiliations:** 1 Department of Urology, Netherland Cancer Institute, Amsterdam, The Netherlands;; 2 Department of Urology, Royal Melbourne Hospital/Peter MacCallum Cancer Centre, Melbourne, Australia

**Keywords:** Prostatic Neoplasms, Prostate cancer, familial [Supplementary Concept], Watchful Waiting, Disease, Therapeutics

## Opinion: Yes

Most men with prostate cancer will not die from it. Although the most frequent cancer in men in the western world it ranks only 3rd place for cause of death (1). In men over 60 years of age prostate cancer is found at autopsy in over 60% (2). Overdiagnosis by PSA screening is estimated to be 57% when screened until 75 years of age (3). Considering treatment toxicity, careful selection of men for treatment is essential. A shift towards more conservative management is apparent in larger registries (4, 5).

In large cohorts of men with biopsy Gleason 6 cancer adverse pathology at prostatectomy is observed in over one-third of men (6, 7) suggesting undersampling of Gleason 7 cancer that is often present in men with Gleason 6 prostate cancer on biopsy. Even in men with very low risk at biopsy (T1c, PSAD<0.15, GS<7, <3 positive cores containing less than 50% cancer), one in 10 will have significant disease at prostatectomy (8).

Reese et al. (9) found the presence of Gleason 7 at biopsy a strong predictor of adverse pathology at prostatectomy. Based on a large prostatectomy series Ploussard et al. (10) concluded that although 46% of men with biopsy Gleason 7 prostate cancer had poor prognostic characteristics at prostatectomy such as upgrading or upstaging at final pathology a subgroup of men with PSA<10ng/ml, PSAD <0.15 cT1c and less than 3 positive cores this risk was only 19% suggesting that in selected men with intermediate risk cancer with otherwise favorable characteristics conservative management can be considered.

Gleason score 7 on biopsy, therefore, does not exert a poorer outcome perse and smaller lesions with this Gleason score may have similar outcome when compared to Gleason 6 prostate cancer on biopsy. The amount of high grade (5) cancer in both biopsy and prostatectomy was found predictive of outcome, rather than Gleason score (11).

These observations show that Gleason 7 on biopsy not necessarily indicates poorer prognosis than Gleason 6 in men with limited Gleason grade 4 disease. Large randomized studies were unable to show a survival benefit of local treatment versus observation in men with Gleason 6 prostate cancer in whom active surveillance is frequently (12). Moreover, in the Pivot trial no difference in survival between active surveillance and prostatectomy was observed for men with intermediate risk prostate cancer. Considering the prognostic overlap between Gleason 6 and 7 prostate cancer on biopsy, men with Gleason 7 prostate cancer could be considered for active surveillance.

The feasibility of active surveillance for intermediate prostate cancer is confirmed by data from Sweden that showed that for men with delayed prostatectomy (median 19m after diagnosis) outcome was similar to early treatment in men with low and intermediate risk prostate cancer (13). Cancer specific survival was 0.7% of men in the primary radical prostatectomy group and 0.9% in men with deferred radical prostatectomy at 8 years follow-up. In a similar report from this registry Stattin et al. report on 93 men with Gleason 7 cancer and active surveillance and 881 with early local therapy consisting of prostatectomy (n=601) or radiotherapy (n=280) (14). Compared to low risk patients, mortality of prostate cancer was twice as high in the intermediate risk group (5.2% versus 2.4%) in active surveillance patients. But the relative risk reduction in the prostatectomy group was less in intermediate risk cancers compared to that of the low risk population (adjusted relative risk in low risk 0.29, intermediate risk 0.53). This observational data suggests that intermediate risk patients were not more likely to be cured by local treatment than low risk patients when compared to active surveillance although the absolute risk of disease specific death in intermediate risk patients is higher.

Several studies on active surveillance did include men with Gleason 7 or intermediate prostate cancer (15). Cooperberg et al. (15) studied outcome in 90 men with intermediate risk prostate cancer and active surveillance. Biopsy Gleason 7 was present in 29 men. Although no difference in survival for low and intermediate risk cancers was found, this study did not report on Gleason 7 intermediate risk cancers separately.

Van den Bergh et al. (16) reported on 50 men with Gleason 7 (6 had Gleason 4+3) prostate cancer managed with active surveillance. Median follow-up was 3.4y. All men with Gleason 4+3 received active treatment compared to 34% of men with Gleason 3+4 suggesting that active treatment rate of Gleason 3+4 is similar to larger low risk active surveillance populations (17).

Longer follow-up is available from the study by Klotz et al. (18, 19). In the initial report, in 72 of 416 men Gleason 3+4 was found at initial biopsy. At a median follow-up of 6.8 years Gleason 3+4 at initial biopsy was a predictor of active treatment, whereas PSA>10ng/ml was not (18). In a recent update from the same institute, Klotz et al. (19) report 993 men on active surveillance with a median follow-up of 6.4 years, shorter than the initial report, but 260 patients within the population had follow-up longer than 10 years. Men over 70 years or a life expectancy less than 10 years and Gleason 3+4 on biopsy and PSA<15ng/ml were allowed to enter. Overall 1.5% died of prostate cancer and 2.8% developed metastases. In the entire population 13% was initially diagnosed with Gleason 7 cancer on biopsy whereas in the men that developed metastases this was 44%. Men with Gleason 7 were 72% more likely to receive active treatment during follow-up, but in the multivariate analysis only Gleason score at one-year rebiopsy and baseline PSA were predictive of active treatment. An important note is the acknowledged limitation of the study that regrading according to current ISUP standards of early cases was not performed, nor were MRI targeted biopsies available at the initial years of inclusion. Both factors suggest that an underestimation of actual Gleason 7 cancer has occurred in men with longer follow-up. Still disease specific survival at 15 years was only 6%, lower than the reported 7% in a large prostatectomy series from the PSA era (20). Patients were 9.2 times more likely to die of other reasons than prostate cancer. It should be noted that patients with an initial Gleason 3+4 were by inclusion criteria more likely to die of non-prostate cancer reasons than low risk men in the study. Additionally, outcome after active local treatment in men with Gleason 6 and Gleason 7 disease treated during follow-up was not different.

Current diagnosis of prostate cancer is often accompanied by MRI imaging (21). Multiparameter MRI (mpMRI) is recommended before starting on active surveillance by the EAU 2016 guidelines for prostate cancer. A recent systematic review (22) revealed that a positive MRI was twice more likely to indicate upgrading on repeat biopsy or prostatectomy. In one-third of men an unrecognised significant lesion may be identified on MRI (23), reclassification is found in 14-20% of men where this lesion is biopsied (23, 24). In men eligible for active surveillance the mpMRI-based PIRADS score improved staging and prediction of upgrading at prostatectomy with a sensitivity of 99% for upgrading and an odds ratio 2.72 in the multivariate prediction. mpMRI with targeted biopsies was shown to increase the detection rate of Gleason 7 cancers while reducing detection of low grade cancers versus repeat random biopsy (25, 26). Due to its low specificity recent smaller series suggest that mpMRI may not replace systematic biopsies in the workup of active surveillance patients (27-29) but may be useful in the follow-up of these men where mpMRI improved the prediction of Gleason progression (30). ADC below median of the population predicted at least a 2-fold higher risk of Gleason progression during follow-up in a population of 86 men with a median follow-up of 9.4 year (31). Whether mpMRI may replace systematic follow-up biopsies is still unclear since in a multivariate analysis mpMRI did not add diagnostic value of high grade cancer to PSA density and biopsy tumor length (32). In the ASIST (Active Surveillance Magnetic Resonance Imaging Study) trial the value of MRI guided confirmatory biopsy in men on surveillance is being studied by the Ontario Institute for Cancer Research and Canadian Urology Research Consortium and data are to be awaited. The use of targeted biopsies will results in the detection of smaller Gleason 3+4 prostate cancers what will make active surveillance in these men more likely.

With respect to including men with Gleason 7 on biopsy and intermediate risk cancer in active surveillance management the following statements should be considered:

- As for GS 6 prostate cancer, no level-1 evidence supports the use of AS for management of GS7/intermediate risk cancers, although randomized studies did not find an advantage of treatment of these cancers.
- In larger series progression to active treatment was found to be twice as high for GS 3+4 compared to biopsy GS 6 cancers.
- Disease upstaging and –grading after active treatment during active surveillance is not more frequent in GS7 compared to GS6 cancers.
- In initial series with longer follow-up tumors may have been undergraded more frequently due to changing Gleason grading criteria (ISUP) and improved detection by MRI. This may have resulted in including a considerable number of men for active surveillance with actual Gleason 7 disease in older series.
- MRI imaging may reveal small Gleason 7 lesions, but with a high certainty of correct risk classification, these may still be amenable to active surveillance.
- Selection for active surveillance should ideally comprise multi-factorial risk estimation, taking into account all tumor and imaging characteristics. With other criteria particularly favorable, Gleason 3+4 or PSA 10-20 may be accepted for active surveillance.

Recommendations from the Canadian Urological Association contain a statement on AS in Gleason 7 disease: “For select patients with low-volume GS 3+4=7 localized prostate cancer, AS can be considered. Survival in men with intermediate risk prostate cancer on active surveillance is high” (33). Although these recommendations are not echoed in the EAU guidelines, the NCCN guidelines now also contain the following phrase: “Patients with favorable intermediate-risk prostate cancer (predominant Gleason grade 3 [i.e., Gleason score 3+4=7], and percentage of positive biopsy cores<50 percent, and no more than one NCCN intermediate risk factor) may be considered for active surveillance” (NCCN website jan. 2016)

Clearly, AS is an option for men with small Gleason 3+4 (but not 4+3). Close follow-up, preferably including mpMRI, is to be considered and men should be informed on the fact that longer term (>10 year) follow-up data are limited.
